# A Physicochemical Study of the Antioxidant Activity of Corn Silk Extracts

**DOI:** 10.3390/foods12112159

**Published:** 2023-05-26

**Authors:** Lubomír Lapčík, David Řepka, Barbora Lapčíková, Daniela Sumczynski, Shweta Gautam, Peng Li, Tomáš Valenta

**Affiliations:** 1 Department of Foodstuff Technology, Faculty of Technology, Tomas Bata University in Zlin, Nam. T.G. Masaryka 275, CZ-762 72 Zlin, Czech Republicsumczynski@utb.cz (D.S.); gautam@utb.cz (S.G.);; 2 Department of Physical Chemistry, Faculty of Science, Palacky University Olomouc, 17. Listopadu 12, CZ-771 46 Olomouc, Czech Republic; david.repka@upol.cz

**Keywords:** corn silk, radical scavenging, electron paramagnetic resonance (EPR), DPPH (1,1-diphenyl-2-picrylhydrazyl), ABTS (2,2′-azino-bis(3-ethylbenzothiazoline-6-sulfonate)) radical cation

## Abstract

Corn silk (CS) extracts are reported to contain flavonoids (appx. 59.65 mg quercetin/g), polysaccharides (appx. 58.75 w.%), steroids (appx. 38.3 × 10^−3^ to 368.9 × 10^−3^ mg/mL), polyphenols (appx. 77.89 mg/GAE/g) and other functional biological substances. This study investigated the antioxidant activity of corn silk extracts related to their functional compounds. The radical scavenging effect of corn silk extracts was evaluated by the spin-trapping electron paramagnetic resonance (EPR) technique, 1,1-diphenyl-2-picrylhydrazyl (DPPH), 2,2′-azino-bis(3-ethylbenzo-thiazoline-6-sulfonate) (ABTS^•+^) free radical measurement, ferric ion-reducing antioxidant power, and copper ion reductive capacity. It was found that the maturity stage of CS plant materials and the applied extraction procedure of their bioactive compounds have a profound effect on the radical scavenging capacity. Differences in the antioxidant activity of the studied corn silk samples based on their maturity were also confirmed. The strongest DPPH radical scavenging effect was observed for the corn silk mature stage (CS-M)stage (CS-MS) (65.20 ± 0.90)%, followed by the silky stage (CS-S) (59.33 ± 0.61)% and the milky stage (CS-M) (59.20 ± 0.92)%, respectively. In general, the final maturity stage (CS-MS) provided the most potent antioxidant effect, followed by the earliest maturity stage (CS-S) and the second maturity stage (CS-M).

## 1. Introduction

Natural products have gained great importance for medicine and food industry due to their effective antioxidant activity. Natural sources such as plants, herbs, fruits, and vegetables contain a variety of antioxidant compounds that can be extracted. In humans, these compounds aid in preventing oxidative stress diseases and counteracting oxidation processes [1]. A reduced risk of cardiovascular disease, cancer, and other chronic diseases is also linked to eating foods high in phenolic and other antioxidant substances. Bioactive compounds possess antioxidant properties through a variety of mechanisms: stabilization of lipid peroxidation is achieved through inhibition of oxidizing enzymes, free radical scavenging activity, and transition-metal-chelating action [2].

Corn silk (*Stigma maydis*) (CS) is a dried thrum and stigma of the female flower *Zea mays* L. ssp. mays, Poaceae (corn plant). It is a cheap, readily available, and high-yielding product that contains many bioactive and functional ingredients [3]. Corn silk has been in human use since time immemorial. In traditional medicine, corn silk was frequently applied as a medicinal herb in drugs and ointments in China, Turkey, the United States, and other countries [4]. Practical applications of corn silk are related to its chemical composition, particularly to the bioactive content of flavonoids (e.g., maysin) [5,6,7], polysaccharides [8], steroids (sitosterol, stigmasterol, etc.), terpenoids, glycoproteins, sterols, alkaloids, vitamins [9,10], and others [11,12]. Corn silk possesses various pharmacological activities, such as antioxidative, antidiabetic, cholagogic, antidepressant and anticancer effects, beneficial to humans [13,14]. It is also used to cure urinary tract infection, malaria, and certain heart diseases [12]. Other health benefits of CS extracts such as diuretic action is associated with its high potassium content [15,16]. Furthermore, corn silk studies also proved corn silk health care values of anti-fatigue [17], and the anti-radiation effect of the CS ethanolic extract on radiation-induced oxidative stress [18]. As reported by Vranješ [19], corn silk extracts could be new therapeutic agents for oxidation- induced kidney diseases.

Despite the aforementioned benefits, corn silk is considered waste from corn processing and is often disposed of, burned, or used as fodder [20]. However, in some countries, corn silk-based products such as tea and powder are now gaining popularity in the market. CS extracts can also be used as an additive in skin creams and cosmetics. Mohsin et al. [21] investigated ethanolic corn silk extracts prepared as water-oil emulsions suitable as an anti-aging cream with antioxidant function. Corn silk has immense potential in future research and commerce in the food industry. It can be consumed as a tea, weight-loss remedy, and as an additive to improve the color and texture of various food products, such as meatballs [22]. Additionally, corn silk fodder (corn scraps) has been successfully used to enhance the nutritional quality of chicken meat [23].

Corn silk flavonoids have potential health benefits due to their antioxidant activity resulting from different conjugation and a varying number of hydroxyl groups present in their chemical structure. Therefore, flavonoids can act as reducing agents, hydrogen- or electron-donating species, and as reactive oxygen species (ROS) scavengers [24,25]. Positive effects of antioxidants correspond to their free radical scavenging ability and with the ability to interact with basic cellular processes [26,27]. Liu et al. (2011) [11] found that specific CS flavonoid glycosides showed significant total antioxidant activity, DPPH radical scavenging activity and reducing power. Žilić et al. (2016) [28] reported that corn silks varieties (yellow, green, pink, and purple colored silks) contained more phenolic and flavonoid compounds and had stronger antioxidant activity in comparison to medicinal herbs (*Mentha piperita*, *Melissa officinalis*, *Ginkgo biloba*, *Thymus serpyllum*, *Salvia officinalis* and green tea). Research of corn silk aqueous acetone extracts by Maksimović et al. (2005) [29] illustrated the polyphenol content as the dominated factor to characterize CS antioxidant capacity.

It was found that the presence of biologically active compounds and related antioxidant properties varied with different CS maturity stages. The composition and concentration of bioactive substances in CS plant materials also depend on biological, pedological, agronomical, and environmental factors [30]. Extraction of bioactive substances from CS materials have been investigated in the last years. Variable techniques of phytochemicals extraction can be involved, including hot water and organic solvent extractions, ultrasonic-, microwave- and enzyme-assisted extractions, and their combinations as well [31,32,33,34,35,36]. Chen et al. (2014) [37] combined enzyme- and ultrasonic-assisted technology of corn silk polysaccharide (CSP) extraction, making extraction conditions milder and more cost-effective. Compared to hot water extraction, the yield of CSP increased, and the enzyme-ultrasonic assisted extracts showed higher antioxidant activities, due to the conformation changes in CSP. In our previous studies, we focused on optimal extraction conditions and extraction yield of corn silk flavonoids [9] and steroids [38].

Due to the shift in consumer preferences to organic foods, the global market has experienced exponential growth in the supply and demand for natural antioxidant products. Therefore, the industrial focus is on the selective extraction of individual biologically active compounds suitable for the production of foods with high antioxidant potency [39,40,41]. Consequently, this brings a great opportunity to develop new products from corn silk materials; many ways can be found to convert corn silk from waste into antioxidant-rich products. Extraction of antioxidative substances from corn silk and their antioxidant activity play a vital role in utilizing CS food and medical supplements. However, there is a lack of studies about the effect of corn silk maturity stages on extraction yield and antioxidant activity of CS extracts, which should be taken into consideration when applying antioxidant tests. This fact was accounted for by the authors of this study. The aim of our research was to analyze antioxidant activity of various corn silk samples in relation to their bioactive compounds profile. The present study deals with the physicochemical characterization of the corn silk bioactive substances prepared at different extraction methods and different CS maturity stages.

## 2. Materials and Methods

### 2.1. Materials

Corn silk (CS) samples were collected from corn kernels produced in a field in the Southern Moravia agricultural region (Uherské Hradiště district, Czech Republic). Three types of corn silk materials were gathered, dependent on the growth stage: the silking stage (CS-S), the milky stage (CS-M), and the mature stage (CS-MS). Fresh corn silk fibers were air-dried for 14 days in the shade. Final drying was performed in a thermostatic hot air-drying oven/sterilizer (Stericell 55 Standard, BMT Medical Technology, Czech Republic). Dried samples were then pulverized in a table-top blender (Philips HR2170/40, The Netherlands) and sifted through a 80 μm mesh sieve (Analysette 3, Fritsch, Germany) to obtain final powder samples [3,42].

All reagents and chemicals used in this study (ethanol, diethyl ether, acetone, 1,1-diphenyl-2-picrylhydrazyl (DPPH), 2,2′-azino-bis(3-ethylbenzothiazoline-6-sulfonate) (ABTS), potassium persulfate K_2_S_2_O_8_, methanol, copper chloride, hydrogen peroxide H_2_O_2_, 5,5-dimethyl-1-pyrroline *N*-oxide (DMPO), vitamin C (VC), rutin, sodium phosphate buffer, potassium ferricyanide K_3_[Fe(CN)_6_], trichloroacetic acid, ferric chloride FeCl_3_, dimethyl sulfoxide (DMSO), neocuproine, and ammonium acetate CH_3_COONH_4_) were of analytical reagent purity grade (Sigma Aldrich, Burlington, MA, USA); distilled water of conductivity 0.6 μS/cm was used as a solvent.

### 2.2. Methodology

#### 2.2.1. Corn Silk Flavonoids Extraction and Determination

To extract flavonoids from corn silk materials, a solid-liquid ratio (1:10) of CS powder and 70 v.% ethanol was used at temperatures of 40 and 80 °C for 20, 30, 40, 50, and 60 min extraction time intervals. Flavonoids extract solutions were centrifuged in Hettich EBA 21 centrifuge (Andreas Hettich GmbH & Co. KG, Tuttlingen, Germany) at 3000× *g* rpm for 10 min to obtain the supernatant [43].

The quantitative determination of flavonoids content was based on rutin standard curve procedure. A UV–Vis spectrophotometer Lambda 25 (Perkin Elmer, Waltham, MA, USA) operating at wavelength 510 nm was used to measure the absorbance of CS extracts and lutin standard solutions prepared by standard determination method [43]. Absorbance vs. concentration dependency data were used to plot the lutin standard curve with linear regression parameters (y = 8.385 (mL/mg).x + 0.007, determination coefficient R^2^ = 0.995) to calculate the content of flavonoids in CS extracts [44].

#### 2.2.2. Corn Silk Polysaccharides Extraction and Determination

To extract polysaccharides, diethyl ether was used to de-grease the CS powder sample at room temperature for 12 h. Then, the powder was dried at a constant temperature of 36 °C in a drying oven; 50 g of dried powder was mixed with 750 mL of distilled water and heated in a water bath to 100 °C for 2 h. Afterward, the samples were centrifuged at 10,000× *g* rpm for 10 min to get the supernatant and sediment. Obtained sediment was used to repeat the aforementioned hot water extraction and centrifugation process. The latter supernatants were then merged and vacuum concentrated (Vacuum rotary evaporator Heidolph Laborota 4010 digital, Heidolph Instruments GmbH & Co. KG, Germany). The concentrated solution was precipitated by 70 v.% ethanol. Obtained sediment was washed with anhydrous ethanol, acetone, and diethyl ether and dried at 50 °C in a drying oven [45].

Glucose standard curve procedure was used to determine the total content of polysaccharides in CS extracts. Glucose standard solutions and CS extracts modified by the standard procedure [45] were measured at wavelength 490 nm. Linear regression parameters of glucose standard curve (y = 3.726(mL/µg).x + 0.0024, R^2^ = 0.998) were plotted to calculate the content of polysaccharides in CS extracts.

#### 2.2.3. Corn Silk Steroids Extraction and Determination

To extract steroids, a solid–liquid ratio CS powder and 70 v.% ethanol (1:20) were used. The mixture was extracted using a 200 W ultrasonic bath (Bandelin Sonorex Super RK 100, Bandelin Electronic GmbH & Co. KG, Germany) for 15, 30, 45, 60, and 75 min at laboratory temperature (25 ± 1 °C). Then, 119 W microwave extraction (apparatus Gallet FMOM 420W, France) was performed for 8 min to obtain CS steroids extracts [45]. Total steroids content in CS extracts was determined by β-sitosterol standard curve procedure. CS extracts and β-sitosterol standard solutions modified by the standard method [45] were measured by UV–Vis spectrophotometry at 530 nm. β-sitosterol standard curve was generated with linear regression parameters (y = 17.82(mL/mg).x + 0.005, R^2^ = 0.999) to calculate the content of steroids in CS extracts.

#### 2.2.4. EPR Spin-Trapping Measurement

The redox system of copper chloride (CuCl_2_) in the presence of hydrogen peroxide under air at 20 °C (293 K) was carried out to generate reactive free radicals to monitor the free radical scavenging capacity (RSC) of studied CS extracts by means of the EPR spin-trapping technique. As a spin-trapping agent 5,5-dimethyl-1-pyrroline *N*-oxide (DMPO) was used.

All EPR measurements were performed in a 4-mm wide flat quartz cell in a Bruker TE_102_ (ER 4102 ST) cavity using an EMX EPR spectrometer (Bruker, Mannheim, Germany) equipped with ER 4111 VT temperature-controlled unit (Bruker, Germany). The reaction mixture was composed of a 50 μL extract in 70% ethanol (50 μL of 70% ethanol was used in a reference experiment), 50 μL of 0.2 M DMPO dissolved in distilled water, and 100 μL of 1 mM CuCl_2_ (in distilled water) and 250 μL of distilled water. Additionally, an analogous set of EPR experiments was performed using CS extracts prepared in distilled water with water as reference. To initiate the copper-ion catalyzed Fenton-like reaction, an aqueous solution of hydrogen peroxide (50 mL of 0.1 M) was added to this mixture and the acquisition of EPR spectra of DMPO adducts started precisely 120 s after H_2_O_2_ addition analogously as described by Jomova et al. [46] and by Simunkova et al. [47]. In order to obtain suitable signal-to-noise ratio, the experimental EPR spectrum represents an accumulation of ten individual 42 s scans. The EPR spin-trapping experiments were carried out in triplicate. The concentrations of DMPO adducts were determined by double integration of the EPR spectra, in relation to the calibration curve obtained from the EPR spectra of Tempol (4-hydroxy-2,2,6,6-tetramethylpiperidine-N-oxyl; Sigma-Aldrich) solutions measured under strictly identical experimental conditions. So evaluated concentration of DMPO adducts after 540 s of CuCl_2_/H_2_O_2_ interaction in solutions containing CS extracts was compared with a reference solution containing ethanol. The observed differences characterize the amount of DMPO adducts scavenged. RSC values were calculated as a percentage of scavenged DMPO adducts relative to aqueous ethanol reference. The experimental EPR spectra of spin adducts were analyzed and simulated using the Bruker software WinEPR (Bruker, Billerica, MA, USA), Winsim2002 (National Institute of Environmental Health Sciences, USA) and EasySpin programs (MATLAB, Natick, MA, USA).

#### 2.2.5. DPPH Assay

A volume of 1 mL of the 70% CS ethanol extract (CS-S, CS-M, and CS-MS) was mixed with 3 mL 1.44 × 10^−4^ mol/L DPPH solution in ethanol; the same concentration of vitamin C and rutin was used as comparison groups. The inhibition level of DPPH was calculated using the following equation:

Inhibition = [(*A*_0_ − *A*_1_)/*A*_0_] × 100% (1)

where *A*_0_ is the absorbance of the control group at the initial time (0 min) and, *A*_1_ is the absorbance of the scavenger sample at the ending time, at 516 nm wavelength [48,49].

#### 2.2.6. ABTS Assay

17.2 mg ABTS was mixed with 3.3 mg K_2_S_2_O_8_ and 5 mL of water as an oxidation starter; ABTS was oxidized to the corresponding radical cations (ABTS^•+^) for 24 h and then kept in the freezer (at −20 °C). Then, 60 mL of distilled water was added to 1 mL of ABTS^•+^ solution to test 70% CS ethanol extract’s radical scavenging activity. 2.5 mL ABTS solution was mixed with 0.5 mL CS extract solutions (each maturity stage of the CS sample was extracted for 60 and 90 min). Vitamin C was used as a comparison at the same concentration as that of CS extract samples; water was used as a reference. Absorbance was measured at 734 nm, and the inhibition level of ABTS^•+^ was calculated by the following equation:

Inhibition = [(*A*_i_ − *A*_e_)/*A*_i_] × 100% (2)

where *A*_i_ is the 734 nm absorbance of the control group at the initial time (0 min) and *A*_e_ is the 734 nm absorbance of the scavenger sample at the ending time [48].

#### 2.2.7. FRAP Assay

Ferric reducing antioxidant power (FRAP) of corn silk extracts and standard materials (vitamin C, rutin) was measured by the standard spectrophotometric method [50,51]. 1 mL of a 70% corn silk extract ethanol solution was put into 2.5 mL 0.2 M phosphate buffer of pH 6.6 and mixed with 2.5 mL 1% K_3_Fe(CN)_6_. The mixture was incubated at 50 °C for 20 min. The reaction was terminated by adding 2.5 mL of 10% trichloroacetic acid followed by mixing of 2.5 mL of upper layer solution with 2.5 mL water and 0.5 mL 0.1% FeCl_3_. Absorbance was measured at 700 nm against a blank sample (DMSO); increased absorbance of mixture reaction represents higher reducing (antioxidant) power.

#### 2.2.8. CUPRAC Assay

Cupric ion-reducing antioxidant capacity (CUPRAC) assay was based on the reduction of copper ions Cu(II) to Cu(I). 0.25 mL of 0.01 M CuCl_2_ was mixed with 0.25 mL of 7.5 × 10^−3^ mol/L neocuproine ethanol solution and 0.25 mL of 1 M ammonium acetate buffer solution, followed by the addition of 0.25 mL of 70% CS ethanol extract. The volume of the mixture was completed to 2 mL by distilled water and maintained at room temperature for 30 min. Absorbance was measured at 450 nm against a blank sample (water); increased absorbance represents the copper ion reductive capability [48,52].

#### 2.2.9. HPLC Polyphenol Analysis

To extract corn silk, a 0.5 g sample (or higher mass if required) was put into a 20 mL aqueous methanol extraction mixture (30:70), shaken at extraction temperatures of 60, 70, or 80 °C for 60 min followed by filtration on 0.2 µm filter membrane.

HPLC analysis of extracted samples was performed by means of De Quiros et al. method [53], with a minor modification [54]. Dionex Ultimate 3000 HPLC system equipped with UV/VIS detector, DAD detector (Thermo Fisher Scientific, Waltham, MA, USA), and LC Chromeleon 7.2 software was used. Chromatographic separation was performed on the Phenomenex Kinetex C18 column (150 × 4.6 mm; 5.0 µm; Torrance, CA, USA) with a sample injection volume of 10 µL.

The analyses were performed in gradient mode, using redistilled water and acetic acid (of 99:1 ratio) as the mobile phase A (99.8%), and redistilled water and acetonitrile and acetic acid (of 67:32:1 ratio) as the mobile phase B (99.8%). Gradient mode was set up in a way to get a constant flow rate of 1.0 mL/min and total dilution time 45 min: decreasing of A to 80% from 0 to 10 min; decreasing of A to 60% from 10 to 16 min; decreasing of A to 50% from 16 to 20 min; decreasing of A to 30% from 20 to 25 min; increasing of A to 90% from 25 to 40 min; and constant at 90% A from 40 to 45 min. Separation was performed at 30 °C column temperature, and chromatograms were detected at 275 nm wavelength. DAD responses were linear for all polyphenol standards used within the calibration range from 0.10 to 150 µg/mL.

#### 2.2.10. Statistical Data Analysis

Observed data were analyzed using a one-way analysis of variance (ANOVA) method and presented as the mean ± standard deviation. Differences in the mean values among statistical groups were tested at significance level α ≤ 0.05. Tukey’s test was applied for multiple comparisons (statistic ranking) of the mean responses to treatment groups (α ≤ 0.05) to evaluate statistical significance, i.e., to determine if they are greater than would be expected by chance. Statistical software SigmaStat version 2.03 (Systat Software, Inc., Palo Alto, CA, USA) was used for data evaluation. All experiments were performed in at least three replicates.

## 3. Results and Discussion

### 3.1. Corn Silk Extraction Process Optimization

#### 3.1.1. Corn Silk Flavonoids Extraction and Determination

The effect of extraction time and temperature (40, 80 °C) was investigated to evaluate the efficiency of flavonoid extraction from CS. All dependencies were of a complex non-linear pattern, modeled as the curves of a third-order polynomial shape, as also confirmed in our previous study [9]. The highest extraction content was found to be 12.2 ± 0.4 µg/mL for CS-MS, and (7.2 ± 0.3) µg/mL for CS-M, extracted for 20 min at 80 °C. However, for the CS-S sample, the highest extraction was determined to be 6.8 ± 2.1 µg/mL, at a combination of 50 min and 40 °C. The extraction trend indicates that the CS flavonoids are temperature sensitive and are likely to undergo thermal degradation at higher temperatures. Similar observations were reported by [55]. Optimal final conditions for flavonoids extraction were characterized as 40 °C for 50 min for the silking stage (CS-S), and 80 °C for 20 min for both the milky (CS-M) and mature stages (CS-MS). Application of the specified conditions provided the most effective CS flavonoids extraction yield.

#### 3.1.2. Corn Silk Polysaccharides Extraction and Determination

**Figure 1:**
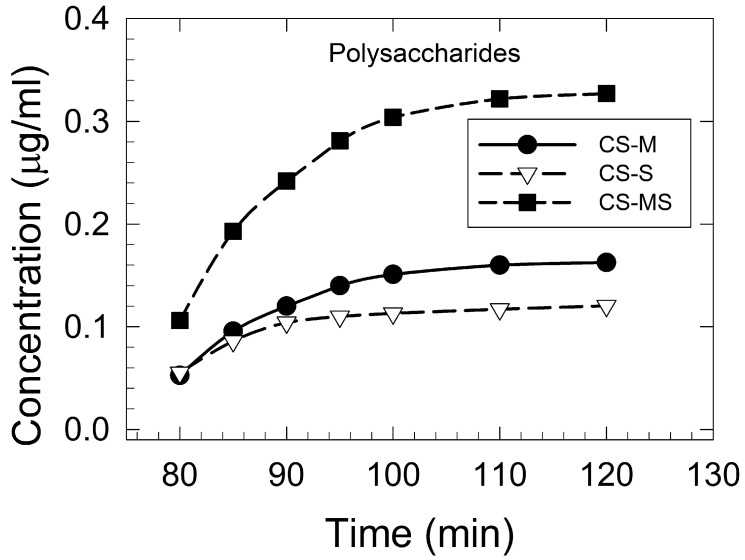
Polysaccharides extraction kinetics (concentration versus time) of corn silk extracts at a 100 °C extraction temperature: the corn silk silking stage (CS-S), the corn silk milky stage (CS-M), and the corn silk mature stage (CS-MS).

Polysaccharide extraction was found to have a positive correlation with extraction time and temperature, especially for the traditional hot water extraction [31]. All the CS maturity samples showed similar exponential extraction kinetics patterns up to a 120 min extraction time (Figure 1). Using a longer extraction time (>120 min) and a higher temperature (>100 °C) had no significant effect on the polysaccharide concentration, indicating 120 min as the optimal extraction time. The highest polysaccharide content was determined for the CS-MS sample (0.33 μg/mL), followed by CS-M (0.16 μg/mL), and CS-S (0.12 μg/mL).

#### 3.1.3. Corn Silk Steroids Extraction and Determination

Steroids extraction kinetics was characterized as a non-linear third-order polynomial dependency [38]. However, there was a proportional trend between corn silk steroid concentration and ultrasonic processing time from 15 to 60 min. In the range of 60–75 min, a slight increase in steroids content was detected. Therefore, 75 min of ultrasonication at laboratory temperature may be defined as the optimal extraction time to gain the maximum steroids content for all CS maturity stages under study, as also confirmed in our previous study [38]. The CS-MS sample yielded the highest steroid concentration (0.37 mg/mL) accompanied with the increased extraction rate via ultrasonic treatment compared to the CS-M sample (0.12 mg/mL) and CS-S (0.09 mg/mL). Based on the above data, the ultrasonic-assisted technique seems to be an effective method for steroids extraction from CS samples, especially from CS-MS.

### 3.2. EPR Spin-Trapping Measurement

**Figure 2:**
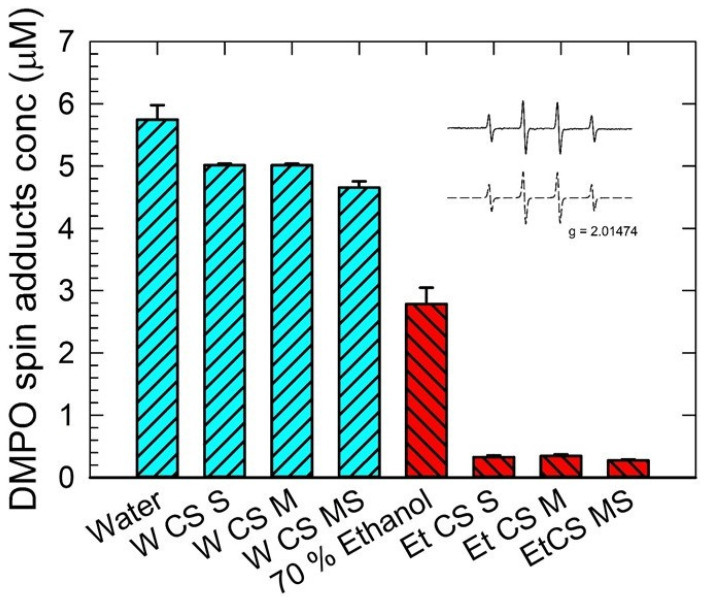
Concentration of •DMPO-OH spin adduct evaluated in CuCl_2_/H_2_O_2_/DMPO solutions containing of corn silk extracts with distilled water (bars with left diagonal lines) and 70% ethanol solvent (bars with right diagonal lines) determined for the silking stage (CS-S), the milky stage (CS-M), and the mature stage (CS-MS). Results are expressed with standard error bars 5% along with the corresponding reference systems. Inset: Experimental (solid line) and simulated (dashed line) EPR spectra of the •DMPO-OH adduct observed as a typical quartet (1:2:2:1) with the spin-Hamiltonian parameters aN = 1.504 mT, aH = 1.484 mT; g = 2.0057.

The EPR spectra monitored in CuCl_2_/H_2_O_2_ solutions containing DMPO spin trap reveal the four-line signal characterized by the spin-Hamiltonian parameters typical for the ^•^DMPO-OH adduct (*a*_N_ = 1.504 mT, *a*_H_ = 1.484 mT; *g* = 2.0057; inset in Figure 2). It was found that the decrease in the ^•^DMPO-OH concentration reflecting the radical scavenging ability of extracts was much stronger for the extracts in 70 v.% ethanol than the ones in water (Figure 2). This indicates the higher efficiency of ethanol as a solvent to extract compounds acting as the radical scavengers. A similar trend in the scavenging ability was observed for the extract series prepared in water or ethanol. It was found that the mature stage (CS-MS) extracts had the strongest radical scavenging ability in comparison to the other stages (CS-M was the weakest). The overall antioxidant activity increased with the maturity of CS, suggesting a higher production of bioactive compounds throughout the natural maturing process.

### 3.3. DPPH Assay

**Figure 3:**
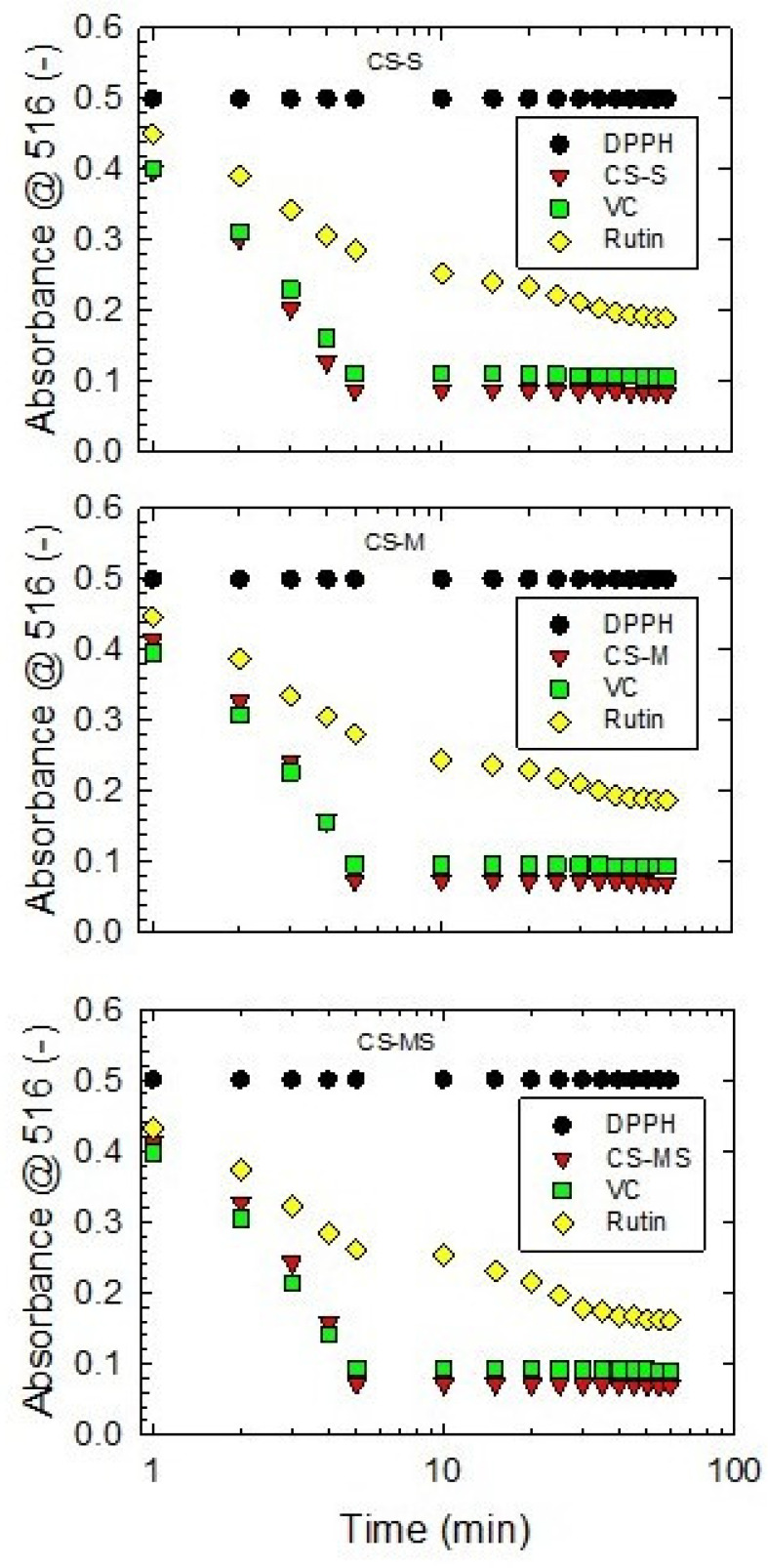
DPPH scavenging kinetics of 70 v.% ethanol corn silk extracts of the silking stage (CS-S), the milky stage (CS-M) and the mature stage (CS-MS) at specified concentrations (0.022 mg/L, 0.029 mg/L and 0.034 mg/L, respectively), compared to vitamin C (VC) and rutin. Absorbance values read at 516 nm wavelength (A_516_) are expressed as the means of three replicates.

CS extracts exhibited a prominent DPPH scavenging effect in the time interval of 0 to 5 min, after which the radical scavenging activity dropped to a constant value (Figure 3). A similar trend was observed for rutin. The radical scavenging activity of CS extracts was found to be more constant than vitamin C. Using a higher CS concentration provided a stronger scavenging effect, consistent with the antioxidant behavior of rutin and vitamin C (Table S1). The values presented are in accordance with the data presented by Abirami et al. [56] for corn silk ethanol extracts. Obtained results are also comparable with data reported by Liu et al. [11] for corn silk fractions extracted in different solvents; the percentage of DPPH radical scavenging activity for CS acetic ether and butanol fractions presented by the authors are in good agreement with the values determined in our study. Moreover, the results of DPPH scavenging activity (Table S1) obtained for different CS maturity stages are compliant with EPR data, as presented in Figure 2. The strongest DPPH radical scavenging effect was observed for the CS-MS sample (65.20 ± 0.90%), followed by CS-S (59.33 ± 0.61) and CS-M (59.20 ± 0.92), respectively. However, the difference between CS-S and CS-M was not statistically significant.

### 3.4. ABTS Assay

**Figure 4:**
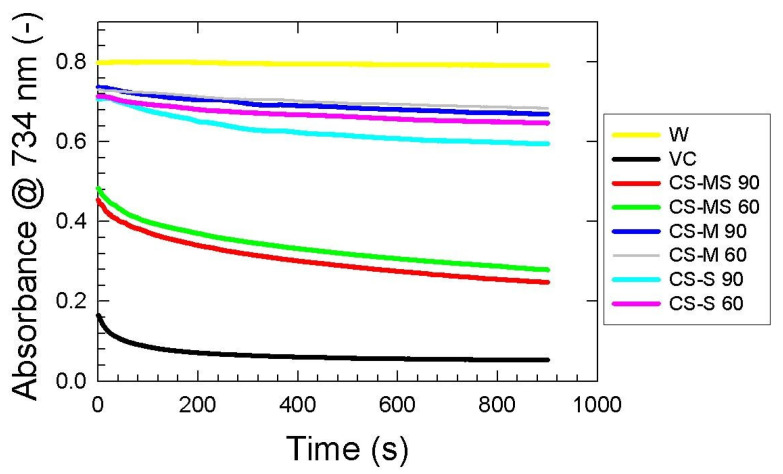
ABTS scavenging kinetics of corn silk extracts dependent on extraction time and maturity stages: the silking stage 60 min (CS-S 60), the silking stage 90 min (CS-S 90), the milky stage 60 min (CS-M 60), the milky stage 90 min (CS-M 90), the mature stage 60 min (CS-MS 60), and the mature stage 90 min (CS-MS 90), compared to vitamin C (VC) and distilled water as reference (W). Absorbance values read at 734 nm wavelength are expressed as the means of three replicates.

It was observed that a longer extraction time for all the CS samples yielded stronger ABTS^•+^ radical scavenging activity (Figure 4 and Table S2). The highest value was found for the corn silk mature stage (CS-MS) sample (65.46 ± 0.72% after 60 min, and 69.17 ± 0.56% after 90 min). However, the radical inhibition activity of the corn silk milky stage (CS-M) (14.52 ± 0.60% and 16.41 ± 0.65% after 60 and 90 min, respectively) was found to be lower than the corn silk silking stage (CS-S) (19.21 ± 0.55% and 25.68 ± 0.60% after 60 and 90 min, respectively), even though the milking stage is more mature than the silking one. This fluctuation of values with respect to the maturity stages was also observed for DPPH scavenging measurement, and can be in relation to the research by [42] who found that water, ethanol and ethyl acetate extracts from immature corn silk can provide a stronger antioxidant capacity than mature CS.

As evident from Figure 4, there is a decreasing trend in absorbance values of corn silk extracts, indicating a progressive reaction between antioxidant compounds and ABTS radicals. After 5 min of this reaction, the trend of scavenging kinetics changes mildly, and the quenching of free radicals becomes less intense. Similar behavior was observed for vitamin C; compared to this substance, CS samples seem to be more time-dependent antioxidants. As expected, inhibition of ABTS radicals is increasing with increasing corn silk extraction times (Supplementary Table S2).

### 3.5. FRAP and CUPRAC Assay

It was observed that the ferric ion-reducing antioxidant power and copper ion reductive capability of CS extracts were dependent on the CS maturity stage (Table 1). The values of ferric ion-reducing study obtained in this study were comparable with the values presented by Liu et al. [11] for corn silk fractions and isolated compounds, investigated by the same method. The CS-MS sample provided the strongest antioxidant effect of FRAP (2.63 ± 0.15), followed by CS-S (1.33 ± 0.32) and CS-M (0.53 ± 0.11). CS-M exhibited the weakest antioxidant capability, which is consistent with the results of previous assays (EPR, DPPH, and ABTS). However, the difference in copper ion reductive capability between CS-S (0.89 ± 0.09) and CS-M (0.78 ± 0.09) was not statistically significant. Moreover, corn silk reducing antioxidant power, as well as thermostability, can be affected by the complexation of CS polysaccharides with Fe^3+^ ions applied in antioxidant assays [13]. Based on the results, it can be assumed that the mature stage of corn silk can provide the strongest antioxidant effect for practical applications and should be one of the key factors to select the corn silk plant material for production.

### 3.6. HPLC Polyphenol Analysis

The polyphenol concentration was determined using HPLC analysis with respect to extraction temperature 60, 70, and 80 °C (Table 2). Results indicate that corn silk (CS) extraction temperature had a significant effect on the concentration of extracted polyphenol substances. This was in agreement with the study published by Jia et al. [31].

The obtained results are shown in Figure S1. Observed patterns were similar for all studied samples (CS-S, CS-M, and CS-MS methanol extracts). Based on the peak analysis, the following chemical substances were identified: caffeic acid, chlorogenic acid, elagic acid, epicatechin, epigallocatechin, ferrulic acid, p-hydroxybenzoic acid, kaempferol, protocatechuic ethylester, protocatechuic acid, rutin, sinapic acid, 2-hydroxycinnamic acid (o-coumaric acid), trans-cinnamic acid, trans-p-coumaric acid, and vanillic acid. Summary of the qualitative and of quantitative analysis data are given in Table 2. As determined by concentration versus temperature extraction kinetics, caffeic acid, protocatechuic ethylester, and trans-p-coumaric acid provided the highest concentration in all CS maturity stages at 70 °C extraction temperature; the highest extraction yield rate at 60 °C was detected for epicatechin, protocatechuic ethylester, and rutin. The other phenolic compounds provided the highest yield rate at 80 °C. Almost all determined polyphenol concentrations significantly correlated with CS maturity stages: CS-MS showed the highest extracted polyphenols content, except vanillic acid. Vanillic acid extraction at 60 °C was found to be 7.37 ± 0.14 μg/g for the milky stage (CS-M) and at 80 °C for the mature stage (CS-MS) to be 88.86 ± 0.44 μg/g. The overall lowest extracted polyphenols content was determined for the silking stage (CS-S). This is probably associated with the fact that the silking stage is the first CS reproductive period, and throughout other stages, most of the bioactive compounds are accumulated in the silk [57]. The present observations are consistent with the findings by Zilic et al. [28], who reported total and specific phenolics concentration to be significantly related to the corn silk maturity stage (harvest time). The authors assessed the silking stage as a rich source of chlorogenic acids, which is consistent with the results of our study: chlorogenic acid and epigallocatechin were detected as the most abundant polyphenols in all CS stages. Moreover, chlorogenic acid concentrations for CS-S are in good agreement with the data reported by Zilic et al. [28], who determined a broad polyphenol concentration range at the silking stage. As also proved by Šukalović [58], the total content of polyphenol substances can be substantially influenced by corn silk (un)pollination, and other factors such as polyphenol oxidase activities may play a role as well [59]. To generalize the results of our study, the corn silk maturity stage was significantly related to the CS polyphenols content, and extraction temperature was significantly influencing CS polyphenol extraction yield rate too.

## 4. Conclusions

This study was conducted to compare different extraction procedures of the bioactive substances from the corn silk material of varying maturity stages. The focus was on identifying and quantifying the flavonoids, steroids, and polysaccharides in the obtained extracts. The following was the antioxidant and radical scavenging activity characterization by selected physicochemical methods. The radical scavenging activity of the extracted substances was measured using EPR, DPPH, ABTS, and other antioxidant measurement techniques. The radical scavenging ability as observed by the EPR spin-trapping method was found to be highest for the mature-stage CS extract (with 70 v.% ethanol) whereas the lowest was found for the milky-stage CS extract. A similar trend was found for DPPH and ABTS radical scavenging activity measurements. A longer extraction time for all the CS samples yielded a stronger ABTS radical scavenging activity. The ferric ion and copper ion-reducing power were found to be highest for the mature-stage CS extract and lowest for the milky stage-CS extract. The results were consistent with the aforementioned results found by the EPR spin-trapping technique, DPPH, and ABTS^•+^. The HPLC polyphenol analysis was performed with respect to temperature. A significant relationship between the extraction temperature and the quantity of obtained polyphenols was found. The fluctuations in the scavenging activity with the increasing maturity stage can be explained by certain developmental and biochemical changes preceding in corn plant during the growth cycle. This variation in CS antioxidant effect should be taken into account when interpreting the results of antioxidant tests. Therefore, further studies with respect to the biochemical changes occurring during the growth cycle and corn silk processing (harvesting, drying, etc.) are of importance. In this context, the effect of corn silk extracts on the human body and the mechanisms of the health benefits provide a great research opportunity. CS extracts have great potential in the food, cosmetics, and nutraceutical industries. The antioxidant nature of CS extracts can be successfully used in developing precision drugs for curing health problems such as hypertension, diabetes, and nephritis.

## Figures and Tables

**Table 1:** Ferric ion-reducing antioxidant power and copper ion reductive capability of corn silk extracts of the silking stage (CS-S), the milky stage (CS-M) and the mature stage (CS-MS).

Corn Silk Sample	Ferric Ion-Reducing Power ^a^	Copper ion Reductive Capability ^a^
CS-S	1.33 ± 0.32 ^a^	0.89 ± 0.09 ^a^
CS-M	0.53 ± 0.11 ^b^	0.78 ± 0.09 ^a^
CS-MS	2.63 ± 0.15 ^c^	1.21 ± 0.13 ^b^

^a^ Data are expressed as the mean ± standard deviation of three replicates. Superscripts with different letters in the same column indicate significant differences between the samples (Tukey’s test, α ≤ 0.05).

**Table 2:** HPLC analysis of polyphenol concentrations in corn silk samples (the silking stage (CS-S), the milky stage (CS-M), and the mature stage (CS-M)stage (CS-MS)) extracted at different temperatures.

Polyphenol	Temperature	Concentration (μg/g) ^a^		
	°C	CS-S	CS-M	CS-MS
Caffeic acid	60	0.74 ± 0.05 ^a^	1.01 ± 0.07 ^ab^	1.51 ± 0.10 ^c^
	70	2.95 ± 0.15 ^d^	4.53 ± 0.18 ^e^	6.22 ± 0.17 ^f^
	80	1.28 ± 0.08 ^bc^	2.26 ± 0.11 ^h^	2.91 ± 0.14 ^d^
Chlorogenic acid	60	6.24 ± 0.23 ^a^	15.21 ± 0.37 ^b^	11.12 ± 0.28 ^c^
	70	22.67 ± 0.30 ^d^	33.55 ± 0.34 ^e^	47.06 ± 0.40 ^f^
	80	97.58 ± 0.52 ^g^	139.67 ± 0.81 ^h^	183.29 ± 1.09 ^i^
Elagic acid	60	17.34 ± 0.28 ^a^	34.71 ± 0.33 ^b^	58.19 ± 0.41 ^c^
	70	17.68 ± 0.25 ^a^	39.02 ± 0.34 ^d^	78.57 ± 0.48 ^e^
	80	28.82 ± 0.31 ^f^	74.16 ±0.44 ^g^	122.69 ± 0.70 ^h^
Epicatechin	60	19.47 ± 0.19 ^a^	23.81 ± 0.29 ^b^	31.23 ± 0.30 ^c^
	70	17.00 ± 0.18 ^d^	20.71 ± 0.25 ^e^	26.37 ± 0.24 ^f^
	80	8.55 ± 0.14 ^g^	10.99 ± 0.21 ^h^	14.04 ± 0.23 ^i^
Epigallocatechin	60	70.70 ± 0.38 ^a^	97.73 ± 0.42 ^b^	137.60 ± 0.97 ^c^
	70	83.39 ± 0.40 ^d^	116.04 ± 0.63 ^e^	162.05 ± 0.65 ^f^
	80	182.12 ± 0.69 ^g^	264.00 ± 1.28 ^h^	343.64 ± 1.55 ^i^
Ferullic acid	60	0.69 ± 0.07 ^a^	1.27 ± 0.10 ^b^	2.38 ± 0.12 ^cd^
	70	0.62 ± 0.09 ^a^	1.65 ± 0.11 ^be^	3.48 ± 0.16 ^f^
	80	0.98 ± 0.08 ^ab^	1.99 ± 0.10 ^ce^	2.75 ± 0.14 ^d^
p-Hydroxybenzoic acid	60	−	−	−
	70	0.30 ± 0.04 ^a^	0.38 ± 0.02 ^a^	0.57 ± 0.05 ^a^
	80	0.39 ± 0.03 ^a^	0.51 ± 0.05 ^a^	0.61 ± 0.04 ^a^
Kaempferol	60	−	−	56.05 ± 0.37 ^a^
	70	34.36 ± 0.33 ^b^	54.60 ± 0.42 ^c^	76.79 ± 0.50 ^d^
	80	76.16 ± 0.49 ^d^	123.11 ± 0.77 ^e^	165.60 ± 0.85 ^f^
Protocatechuic ethylester	60	4.71 ± 0.17 ^a^	14.43 ± 0.16 ^b^	23.60 ± 0.22 ^c^
	70	14.41 ± 0.11 ^b^	23.31 ± 0.18 ^c^	33.93 ± 0.28 ^d^
	80	4.11 ± 0.09 ^e^	6.99 ± 0.12 ^f^	9.62 ± 0.10 ^g^
Protocatechuic acid	60	0.61 ± 0.08 ^a^	0.82 ± 0.10 ^a^	1.32 ± 0.12 ^b^
	70	0.33 ± 0.07 ^c^	0.77 ± 0.11 ^a^	1.22 ± 0.14 ^b^
	80	0.29 ± 0.09 ^c^	0.64 ± 0.10 ^a^	0.90 ± 0.12 ^a^
Rutin	60	−	7.49 ± 0.13 ^a^	10.79 ± 0.16 ^b^
	70	3.32 ± 0.17 ^c^	4.79 ± 0.11 ^d^	6.48 ± 0.18 ^e^
	80	−	−	−
Sinapic acid	60	0.10 ± 0.02 ^a^	−	−
	70	−	−	−
	80	1.91 ± 0.08 ^b^	2.77 ± 0.14 ^c^	4.15 ± 0.13 ^d^
trans-2-Hydroxycinnamic acid	60	3.59 ± 0.11 ^a^	4.67 ± 0.15 ^b^	7.13 ± 0.18 ^c^
	70	2.88 ± 0.09 ^d^	4.46 ± 0.10 ^e^	6.90 ± 0.15 ^c^
	80	7.63 ± 0.16 ^f^	11.89 ± 0.18 ^g^	16.88 ± 0.13 ^h^
trans-Cinnamic acid	60	−	−	8.48 ± 0.13 ^a^
	70	5.20 ± 0.15 ^b^	6.20 ± 0.19 ^c^	11.62 ± 0.22 ^d^
	80	11.53 ± 0.24 ^d^	18.64 ± 0.27 ^e^	25.07 ± 0.30 ^f^
trans-p-Coumaric acid	60	2.21 ± 0.14 ^a^	3.98 ± 0.06 ^b^	6.16 ± 0.10 ^c^
	70	5.76 ± 0.17 ^d^	9.36 ± 0.15 ^e^	15.21 ± 0.22 ^f^
	80	4.87 ± 0.16 ^g^	7.68 ± 0.11 ^h^	10.36 ± 0.15 ^i^
Vanillic acid	60	3.02 ± 0.09 ^a^	7.37 ± 0.14 ^b^	5.39 ± 0.12 ^c^
	70	10.99 ± 0.15 ^d^	16.26 ± 0.20 ^e^	22.81 ± 0.16 ^f^
	80	47.31 ± 0.23 ^g^	67.71 ± 0.38 ^h^	88.86 ± 0.44 ^i^

^a^ Data are expressed as the mean ± standard deviation of three replicates. Superscripts with different letters for concentrations of the polyphenol in the same paragraph indicate significant differences between the values (Tukey’s test, α ≤ 0.05). The hyphen means that no value was detected.

## Data Availability

The datasets generated in this study are available on request to the corresponding author.

## Supplementary Materials

The following supporting information can be downloaded at: https://www.mdpi.com/article/10.3390/foods12112159/s1, Table S1: DPPH radical scavenging activity of corn silk extracts: the silking stage (CS-S), the milky stage (CS-M) and the mature stage (CS-MS), in comparison to vitamin C (VC) and rutin. Table S2: ABTS free radicals inhibition by corn silk extracts of the silking stage (CS-S), the milky stage (CS-M) and the mature stage (CS-MS) dependent on their extraction time, in comparison to vitamin C (VC). Figure S1: HPLC chromatograms of corn silk maturity stages (the silking stage (CS-S), the milky stage (CS-M), and the mature stage (CS-M)stage (CS-MS)) in aqueous methanol solutions extracted at different temperatures (60 °C, 70 °C and 80 °C). Total elution time was 45 min.
