# Supplementary material for: A Physicochemical Study of the Antioxidant Activity of Corn Silk Extracts
Source: Foods. 2023 May 26;12(11):2159. doi: 10.3390/foods12112159 (PMC10252499; doi:10.3390/foods12112159)
Supplement: Supplementary file 1 [file foods-12-02159-s001.zip › foods-2385885-supplementary.docx]

Supplementary Data

Physicochemical study of the antioxidant activity of the corn silk extracts

Lubomír Lapčík ^1,2^, David Řepka ^2^, Barbora Lapčíková ^1,2^, Daniela Sumczynski ^1^, Shweta Gautam ^1^, Peng Li ^1^ and Tomáš Valenta ^1^

^1^ Tomas Bata University in Zlin, Department of Foodstuff Technology, Faculty of Technology, Nam. T.G. Masaryka 275, 762 72 Zlin, Czech Republic

^2^ Palacky University Olomouc, Department of Physical Chemistry, Faculty of Science, 17. Listopadu 12, 17. Listopadu 12, 771 46 Olomouc, Czech Republic

***** Correspondence: lapcikl@seznam.cz.

**Table S1.** DPPH radical scavenging activity of corn silk extracts: silking stage (CS-S), milky stage (CS-M) and mature stage (CS-MS), in comparison to vitamin C (VC) and rutin.

| **Scavenger** | **Concentration (mg/ml)** | **Inhibition of DPPH (%)^a^** |
| --- | --- | --- |
| CS-S | 0.22 | 59.33 ± 0.61 a |
| VC |  | 78.63 ± 0.45 b |
| Rutin |  | 62.03 ± 0.35 c |
| CS-M | 0.29 | 59.20 ± 0.92 a |
| VC |  | 80.63 ± 0.96 bd |
| Rutin |  | 62.30 ± 0.76 c |
| CS-MS | 0.34 | 65.20 ± 0.90 e |
| VC |  | 81.63 ± 0.67 d |
| Rutin |  | 67.30 ± 1.08 e |

^a^ Data are expressed as mean ± standard deviation of three replicates. Superscripts with different letters in the same column indicate significant differences between the samples (Tukey test, α ≤ 0.05).

**Table S2.** ABTS free radicals inhibition by corn silk extracts of silking stage (CS-S), milky stage (CS-M) and mature stage (CS-MS) dependent on their extraction time, in comparison to vitamin C (VC).

| **Scavenger** | **Extraction time (min)** | **Inhibition of ABTS (%)^a^** |
| --- | --- | --- |
| CS-S | 60 min | 19.21 ± 0.55 a |
| CS-M |  | 14.52 ± 0.60 b |
| CS-MS |  | 65.46 ± 0.72 c |
| CS-S | 90 min | 25.68 ± 0.60 d |
| CS-M |  | 16.41 ± 0.65 e |
| CS-MS |  | 69.17 ± 0.56 f |
| VC | - | 93.28 ± 0.45 g |

^a^ Data are expressed as mean ± standard deviation of three replicates. Superscripts with different letters in the same column indicate significant differences between the samples (Tukey test, α ≤ 0.05).


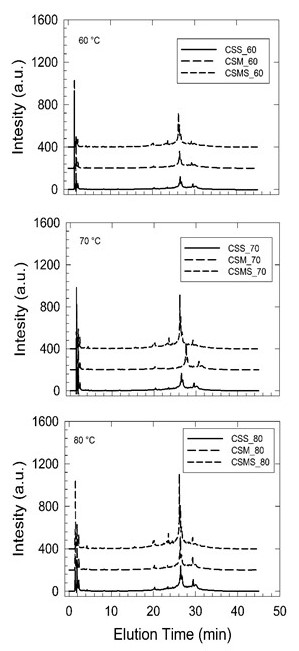


**Figure S1.** HPLC chromatograms of corn silk maturity stages (silking stage CS-S, milky stage CS-M, and mature stage CS-MS) in aqueous methanol solutions extracted at different temperatures (60 °C, 70 °C and 80 °C). Total elution time was 45 min.
